# Peripartum COVID-19 & hemophagocytic lymphohistiocytosis: a case report

**DOI:** 10.1515/crpm-2024-0033

**Published:** 2024-12-17

**Authors:** Kathy Mostajeran, Daniel Rabulinksi, Abdul Khan, Nehan Sher, Christopher K. Huls, Chien C. Oh

**Affiliations:** Department of Obstetrics and Gynecology – Maternal-Fetal Medicine, University of Arizona College of Medicine/Banner University Medical Center, Phoenix, AZ, USA; Department of Pulmonary & Critical Care Medicine, University of Arizona College of Medicine/Banner University Medical Center, Phoenix, AZ, USA

**Keywords:** hemophagocytic lymphohistiocytosis, puerperal, pregnancy, COVID-19

## Abstract

**Objectives:**

Hemophagocytic lymphohistiocytosis (HLH) is a rare disorder of hypercytokinemia and immune dysregulation. Most commonly diagnosed in the pediatric population due to genetic predisposition, the condition can manifest in the adult population secondary to an immune dysregulating event, such as infection, malignancy, rheumatologic disorders, immunodeficiency, and checkpoint inhibitors. The presentation and diagnosis during pregnancy are extremely rare and elusive. We present a case of secondary HLH during the peripartum period, urging obstetrical providers to keep the condition as part of their differential diagnosis.

**Case presentation:**

A 20-year-old Gravida 1, with a past medical history significant for non-alcoholic hepatosteatosis and morbid obesity, presented multiple times to the emergency department in the third trimester with liver function test derangements and vague complaints of subjective fevers and fatigue. She eventually tested positive for COVID-19. Two weeks after the initial presentation, she went into spontaneous preterm labor and delivered. Postpartum, her liver dysfunction worsened in association with high fevers and persistent tachycardia. After an extensive workup failed to reveal an etiology, HLH was suspected. Labs were sent for confirmation, and she was initiated on pulse-dose steroids. However, the patient acutely decompensated and succumbed to the disease. Several days later, labs resulted, confirming the diagnosis of HLH.

**Conclusions:**

In peripartum patients presenting with severe derangements in liver function tests and vague symptoms with undulating episodes of pyrexia, HLH should be considered early as part of the differential diagnosis. This is particularly true when antibiotics or postpartum status fail to alleviate the symptomatology or improve the clinical course.

## Introduction

Hemophagocytic lymphohistiocytosis (HLH) is an inflammatory disorder in which the immune system is excessively activated. This results in dysregulation and tissue damage, leading to further immune activation and tissue destruction. Thus, a vicious cycle ensues, with a markedly elevated risk of morbidity and mortality, especially when treatment is delayed.

Primary HLH, often caused by mutations of genes encoding for components in the mechanism of perforin-dependent cytotoxicity, manifests early in life within the pediatric patient population. Secondary HLH, which is more common in adult patients, is triggered most often by an infectious etiology, although other factors such as malignancy, rheumatological disorders, immunodeficiency, and the use of checkpoint inhibitors have also been implicated. Viral exposures are one of the more commonly cited HLH triggers in the literature, specifically the Epstein-Barr virus (EBV) and, more recently, the COVID-19 virus (SARS-CoV-2).

HLH clinically manifests as an acute or subacute febrile syndrome with multi-organ involvement. Signs typically can include hepatosplenomegaly, hepatic dysfunction, cytopenia of two or three lineages, hyper-ferritinemia, hypertriglyceridemia, and/or hypofibrinogenemia. Making the diagnosis during pregnancy is often challenging due to the considerable overlap of both sepsis and pregnancy-specific disorders such as acute fatty liver of pregnancy and hemolysis, elevated liver enzymes, and low platelets (HELLP) syndrome. It is crucial to include HLH within the differential diagnosis of these disorders, particularly in the setting of pyrexia and negative blood cultures, as delay in HLH-specific treatment delay often leads to high mortality in this population.

We present a case of peripartum HLH due to SARS-CoV-2 in a patient in which pregnancy complicated the clinical scenario.

## Case presentation

A 20-year-old Gravida 1 presented to the emergency department multiple times between 32- and 34-weeks gestational age before ultimately being admitted for preterm labor to the antepartum unit. The patient’s past medical history was significant for morbid obesity (body mass index (BMI) 65 kg/m^2^) and a reported prior diagnosis of non-alcoholic hepatosteatosis; however, her prenatal care had been uncomplicated to date. Vital signs and labs are noted for the three emergency department visits prior to her antepartum admission in Table 1. With each subsequent visit, her complaints progressed. Her initial complaint included four days of waxing and waning fevers associated with weakness. At that time, tachycardia was present, and labs were notable for elevated liver enzymes (AST=779 U/L; ALT=876 U/L). Test results for RSV, SARS-CoV-2, and Influenza were negative. A right upper quadrant ultrasound was performed, and the findings suggested steatohepatitis. At the second emergency room visit, she complained of ongoing persistent fevers now associated with dyspnea with exertion. A computed tomography of the chest utilizing a pulmonary embolism protocol was unremarkable. Labs were notable for a white blood cell count of 4.0 K/µL and persistently elevated liver enzymes (AST=606 U/L; ALT=470 U/L). Upon presentation for the third time, she complained of 10 days of waxing and waning fevers, worsening dyspnea, which was now present at rest, and a new onset cough. The patient tested positive for SARS-CoV-2. She was given intravenous methylprednisolone and discharged home. Of note, her lactic acid was never greater than 2.0 mmol/L at any emergency department visit.

**Table 1:** Vital signs and lab values throughout the patient’s clinical course. Daily averages are presented as a single number with numbers in parentheses indicating the range of values for that 24-h period. “✕” represents a lack of available labs during that specific 24-h period. “-” represents a negative test result, while “+” represents a positive test result.

	Day 1	Day 4	Day 9	Days 14 to 16 (antepartum)	Day 17 (delivery)	Day 18	Day 19 (transported)	Day 20	Day 21	Day 22
Temperature, °C	37.6	37.5	37.0	37.2 (36.4 °C–37.6 °C)	38.0 (36.5–39.8)	36.8 (35.3–38.5)	36.5 (35.5–38.3)	39.2 (38.6–39.8)	40.4 (38–42.5)	37.0 (36.5–37.4)
Heart rate, bpm	138	123	105	116 (98–134)	147 (116–159)	111 (90–169)	128 (110–176)	152 (140–166)	139 (113–171)	128 (113–147)
Respiratory rate	16	20	33	23 (18–32)	35 (20–44)	31 (19–53)	31 (15–44)	34 (22–60)	32 (16–42)	30 (30–30)
SARS-CoV-2	–	–	+	✕	✕	✕	✕	✕	–	✕
WBC, K/µL	6.4	4.0	4.4	8.0	8.1	4.3 (3.3–6.4)	3.6 (3.0–4.2)	8.45 (4.9–12.0)	11.3 (7.9–14.5)	13.6
Hemoglobin, g/dL	11.1	10.5	9.8	10.6	10.7	9.0 (8.5–10.2)	9.8 (9.8–9.8)	8.9 (8.2–9.6)	9.2 (8.7–9.6)	9.5
Platelets, K/µL	265	269	315	243	234	111 (75–143)	103 (88–129)	108 (102–113)	96 (67–109)	43
AST, U/L	779	606	408	598	756 (686–825)	1,236 (764–1,905)	1,904 (1,687–2,139)	1,795 (1,594–2,225)	1,996 (1,601–2,282)	6,168
ALT, U/L	876	470	233	190	219 (209–228)	279 (204–384)	402 (367–418)	379 (343–454)	380 (348–412)	689
Total bilirubin, mg/dL	1.6	2.0	2.1	✕	1.45 (1.4–1.5)	2.0 (1.3–2.7)	2.8 (2.4–3.3)	3.95 (3.6–4.5)	4.8 (4.4–5.2)	5.1
INR	✕	✕	✕	✕	1.1	1.3	1.5 (1.3–1.6)	1.4 (1.3–1.5)	1.9 (1.4–3.1)	✕
Fibrinogen, mg/dL	✕	✕	✕	✕	✕	✕	57 (38–75)	94 (65–121)	78 (42–96)	✕
Triglycerides, mg/dL	✕	✕	✕	✕	✕	✕	✕	1,513	1,219 (858–1,579)	763
Ferritin, ng/mL	✕	✕	✕	✕	✕	✕	✕	23,175	✕	✕
Lactic acid, mmol/L	1.2	1.2	1.9	✕	9.6 (8.7–10.5)	6.0 (5.5–6.7)	7.9 (7.1–9.4)	9.0 (7.5–10.5)	6.2 (4.9–7.0)	14.5
Serum creatinine, mg/dL	0.46	0.44	0.44	0.37	0.53 (0.50–0.55)	0.39 (0.35–0.45)	0.35 (0.29–0.45)	0.41 (0.35–0.45)	0.54 (0.044–0.83)	0.67
Blood cultures	–	✕	–	✕	–	✕	–	✕	–	✕

The patient presented for a fourth time 13 days after initial presentation. The patient complained of abdominal pain and vaginal bleeding and was admitted for preterm labor at 34 weeks gestation. She still complained of persistent dyspnea and fevers but was oxygenating well on room air without supplemental oxygen. She received standard care, including betamethasone for late preterm antenatal corticosteroid treatment. She was also treated with acetaminophen for her fever, which was no more than 1 g per day. Due to the concern for a urinary tract infection, she was started on daily ceftriaxone on admission, although urine cultures were negative. After two days of uterine quiescence, her amniotic membranes spontaneously ruptured. The patient was switched to intravenous penicillin for group beta-strep prophylaxis and underwent an uncomplicated spontaneous vaginal delivery with a small left labial laceration repaired in a routine fashion.

Five hours after delivery, internal medicine was consulted for persistent tachycardia. An electrocardiogram demonstrated sinus tachycardia. As noted in Table 1, temperature ranged from 36.5 °C to a maximum of 39.8 °C throughout postpartum day zero. Antibiotics were expanded to cefepime. Eventually, she became slightly hypotensive but responded to fluid administration. She was upgraded to the intensive care unit for suspected septic shock. Antibiotic therapy was broadened to include piperacillin/tazobactam, and blood cultures were repeated. Tylenol was discontinued and N-acetyl cysteine (NAC) was also initiated at this time. Labs are shown in Table 1, most notable for elevated liver enzymes and elevated lactic acid. Similar temperature ranges are noted on postpartum day one as well as worsening liver enzyme function testing, hypoglycemia that responded to IV dextrose administration, and a slight decrease in the lactic acid (from an average of 9.6 mmol/L to 6.0 mmol/L). On postpartum day one, the decision was made to transfer the patient for a higher level of care.

The patient arrived at our hospital on postpartum day two with clinical findings significant for anasarca, respiratory distress, tachycardia, and persistent fevers. On genitourinary examination, the previous left labial laceration was healing well; however, severe tenderness on bimanual was noted. The clinical diagnosis was postpartum endometritis, and antibiotics were changed to ampicillin, gentamicin, and clindamycin per obstetrical guidelines. Computed tomography of the abdomen and pelvis with contrast was significant for hepatosplenomegaly, abdominal ascites, and complex echogenic material in the lower uterine segment with concern for possible retained products of conception. Findings were consistent with a transvaginal ultrasound. A duplex ultrasound of the liver was also performed, which was significant for hepatic steatosis with a limited examination of the flow due to habitus. The results of a comprehensive hepatology panel, autoimmune panel, and viral panel, including EBV, were negative. See Table 2 for the complete list of labs drawn.

**Table 2:** List of pertinent labs drawn and results during admission.

Laboratory study	Result
WBC-flow cytometry	8,626 Cells/uL
Lymphs, absolute-flow cytometry	2,973 Cells/uL
CD 56 (absolute)	12.9 % (384/uL)
Autoimmune panel^a^	Negative
Anti-nuclear antibodies	Negative
Anti-mitochondrial antibodies	Negative
Smooth muscle antibodies	Negative
Anti-microsomal liver/kidney antibodies	Negative
Anti-double stranded DNA antibody index	<1.0 IU/mL (negative)
Myositis panel^b^	Negative
Iron	135 ug/dL
Ceruloplasmin	27 mg/dL
Alph-1-antitrypsin	202 mg/dL
IgG	273 mg/dL
Epstein barr virus, by PCR	Not detected
EBV, nuclear Ag, EBNA	77.10 U/mL
VC IgG/IgM	118.00 U/mL/<36.00 U/mL
Cytomegalovirus DNA, PCR	<35 IU/mL
CMV IgG/IgM	6.3 AI/<0.2 AI
Hepatitis a ab, Total/IgM ab	Reactive/non-reactive
Hep B surface antibody result	<3.50 mIU/mL
Hepatitis B surface antigen	Non-reactive
Hepatitis B core ab, total	Non-reactive
Hepatitis C ab screen	Non-reactive
Hepatitis E IgM/IgG	Not detected/Not detected
Quantiferon (TM), NIL	0.86 IU/mL
Coccidioides EIA ab, IgM/IgG	Negative/Negative
RPR	Non-reactive
HIV 1/2 A g/Ab screen (4th gen)	Non-reactive

^a^Autoimmune panel included all of the following labs. ^b^Myositis panel includes all the following: SSA 52 (Ro) (ENA) Antibody IgG, SSA 60 (Ro) (ENA) Antibody IgG, Smith/RNP (ENA) Ab, IgG, Jo-1 (histidyl-tRNA synthetase) Ab, IgG, PL-12 (alanyl-tRNA synthetase) Antibody, PL-7 (threonyl-tRNA synthetase) Antibody, EJ (glycyl-tRNA synthetase) Antibody, OJ (isoleucyl-tRNA synthetase) Antibody, SRP (signal recognition particle) Ab, Ku Antibody, PM/SCL 100 Antibody, IgG, Fibrillarin (U3 RNP) Ab, IgG, Mi-2 (nuclear helicase protein) Antibody, P155/140 Antibody, TIF-1 gamma (155 kDa) Ab, SAE1 (SUMO activating enzyme) Ab, MDA5 (CADM-140) Ab, NXP2 (nuclear matrix proten-2)Ab.

On postpartum day three, the patient was taken to the operating room due to a lack of clinical improvement with antibiotic therapy although multiple sets of blood cultures previously drawn were negative. In the operating room, an external genitourinary examination did not reveal any evidence of purulent discharge or necrosis, and the previous left labial laceration appeared to be healing well. Dilation and curettage was performed under ultrasound guidance. The endometrial stripe appeared thin, little tissue was obtained, and excellent uterine resistance was noted with a low suspicion for endomyonecrosis. Simultaneously, a culdocentesis was performed to collect ascitic fluid. Both peritoneal fluid and endometrial tissue were sent for gram stain and culture. The results of both samples were negative on gram stain and did not reveal any underlying infectious etiology. A histological review of the endometrial tissue was negative for retained products of conception or acute inflammation. During the procedure, she received norepinephrine to support her blood pressure and was started on propofol and fentanyl for analgesia and sedation. In addition, she received 100 mg of intravenous hydrocortisone.

Given her clinical condition postoperatively, the patient returned to the intensive care unit, remained intubated, sedated, and on minimal pressor support. Of note, when a sedation pause was performed at this time, the patient was alert and answering questions appropriately. As part of the protocol for propofol use in the ICU, serum triglycerides were checked and noted to be 1,513 mg/dL, for which an insulin drip was then initiated. There was a concern for possible HLH, and a ferritin level was acquired, which measured 23,173 ng/mL. She improved throughout the morning on postpartum day four (postoperative day 1), but by early evening, the patient began to have increased pressor requirements and persistent hypoglycemia despite stopping insulin drip and IV dextrose administration and her mental status became more altered. A transthoracic echocardiogram was performed which showed a left ventricular ejection fraction greater than 70 %, normal right ventricular systolic function, negative bubble study, and a right atrial pressure of 15  mmHg. Her pressor requirements continued to escalate, and maximal doses of norepinephrine, vasopressin, and phenylephrine were reached. Soluble IL-2 receptor and CXCL9 were collected earlier but required processing at an outside laboratory. She developed renal failure alongside her worsening shock and she was initated on continuous renal replacement therapy. The patient was given a pulse dose of 500 mg of IV methylprednisolone with the plan for a bone marrow biopsy the next day prior to initiating HLH-specific treatment. Unfortunately, the next morning, on postpartum day five, she rapidly declined and demised. Labs resulted 48 h after the patient’s death, which demonstrated a soluble IL-2 receptor 11,735 U/mL and a CXCL9 value of >608,000 pg/mL, which secured the diagnosis of HLH.

## Discussion

Hemophagocytic lymphohistiocytosis is an exceedingly rare condition to present during pregnancy but can present at any gestation and the postpartum period. A review of reported case studies by Lui et al. systematically analyzed 81 cases of HLH diagnosed during pregnancy or postpartum [1]. Only 51 patients demonstrated a clear etiology, with infection being the most prevalent [1]. Researchers found that the presentation during the antepartum (80 % of cases) is often nonspecific, with the most common presenting complaint being persistent or intermittent fevers (69 %) [1]. Markedly elevated liver enzymes have been associated with the presentation of HLH during pregnancy, with a review of seven cases in the United Kingdom reporting 71 % of patients presenting with such lab abnormalities [2].

Our patient presented to an outside hospital’s emergency room multiple times with complaints of intermittent fevers almost two weeks before admission for preterm contractions, consistent with a prodromal period. After the initial presentation, she was diagnosed with SARS-CoV-2 and received an intravenous dose of methylprednisolone in the emergency department about five days before admission for preterm labor. During these visits, it was also noted that she had waxing and waning liver enzymes that were attributed to a viral upper respiratory syndrome due to her concurrent complaint of dyspnea, tachycardia, and intermittent fevers. It should be recognized that SARS-CoV-2 has been associated with transaminitis in pregnancy and has been found to be associated with increased severity of the disease [3]. The differential diagnosis of pregnancy associated and pregnancy specific conditions with overlapping features is presented in Table 3.

**Table 3:** Differential diagnosis of elevated liver enzymes in pregnancy and postpartum period; comprised of both expert knowledge and percentages specifically from references [1, 2, 6] where appropriate has been referenced within the table.

	HLH (peripartum specific)	HELLP	AFLP	Intrahepatic cholestasis
Incidence	Extremely rare	0.5–0.9 %	0.005–0.01 % [4]	0.1 % (USA)
Time of presentation	Any trimester & postpartum	70 % 3rd trimester 30 % postpartum	Late 2nd-3rd trimester	Late 2nd-3rd trimester
Presenting symptoms	Prolonged duration of fevers; upper URI symptoms, lymphadenopathy, pruritus rash, nausea, jaundice	RUQ or epigastric pain	1–2-week prodrome nausea, vomiting, anorexia, malaise; polyuria/polydipsia (5 %)	Pruritus particularly worse at night involving the palm and soles; jaundice (∼10–25 %)
Fever	++ high grade or intermittent (69 %) [2]	–	+/−often low grade if present	–
Ascites	+	–	+	–
Hepatomegaly	+/−	+/−	+/−	–
Splenomegaly	+ (100 %) [2]	–	–	–
Hemolysis	–	+	+/−	–
Platelets, ×10⁹/L	↓↓↓ ≤100	↓↓ <150	↓ ≥100	↓/↔ >100
Leukocytes, K/µL	↓ ≤4,000 (most common)	↔	↑ ≥11,000 (100 %) [4]	↔
Liver enzymes, U/L	↑↑↑ 5–30 × upper limit	↑ ≤ 500	↑↑↑ 2–100 × upper limit	↑ 1–5 × upper limit
Triglycerides, mg/dL	↑↑↑	↔	↔	↑
Total bilirubin, mg/dL	↑↑↑ ≥5 but often >10 [1, 2]	↑ <5	↑↑ <10	↑ <5
Ferritin, ng/mL	↑↑↑ >500, but often >5,000 [1, 2]	<500	<500	↔
Fibrinogen, mg/dL	↓↓↓	Slight ↓/↔ (>200)	Slight ↓ (<250)	↔
DIC	+	+/−	+/−	–
Acute kidney injury	Uncommon	–	Creatinine >1.0 mg/dL (96 %) [4]	–
Resolution with delivery	Possibly? Suggested by some case reports [2]	Yes, most demonstrate clinical recovery 2–4 days postpartum	Yes, most demonstrate clinical recovery 3–4 days postpartum	Yes, commonly 1–2 days postpartum, sometimes 1–2 weeks

HLH, hemophagocytic lymphohistiocytosis; HELLP, hemolysis, elevated liver enzymes, low platelets; AFLP, acute fatty liver of pregnancy.

We theorize that our patient’s immune dysregulation was initially triggered by SARS-CoV-2, which stimulated her preterm labor with rapid delivery. With the process of parturition itself being a self-propagating hyperinflammatory process, delivery resulted in a slight improvement in her symptoms postpartum. However, given the indolent course and long duration of her prodromal period, we suspect that the process of inflammatory dysregulation had far progressed. While delivery has resolved HLH in some patients, it served as only a brief temporizing measure for our patient [1]. Without immediate intervention with immunosuppressive treatment, the patient died after a rapid deterioration. The patient’s rapid progression limited our ability to safely exclude an infectious viral etiology as the underlying mechanism prior to initiating immunosuppressive treatment, which resulted in delayed treatment.

The literature is conflicting with respect to SARS-CoV-2 as a trigger for secondary HLH, in part due to literature demonstrating that many patients with severe SARS-CoV-2 and immune dysregulation did not achieve the diagnostic criterion of HLH based on published scoring systems [5]. However, our patient met HLH 2004 diagnostic criteria as well as had an H-score of 269, suggesting a >99 % likelihood of HLH [6]. While we were unable to undertake genetic analysis, the only potential explanation as a trigger was the concurrent diagnosis of SARS-CoV-2 during her prodromal symptoms. After reviewing the available literature, we were unable to find a case report tying SARS-Cov-2 as a stimulus for secondary HLH occurring during pregnancy, making this the first reported case.

Our takeaway from this complicated and challenging case is that HLH be considered early as part of the differential diagnosis in pregnant and postpartum patients who present with significantly abnormal liver enzymes and vague symptoms in the setting of fevers. A high index of suspicion for HLH, especially in the setting in which antibiotics and delivery fail to improve the clinical course, is important in diagnosing this challenging entity and preventing high morbidity and mortality. 
